# The role of B cells in heart failure and implications for future immunomodulatory treatment strategies

**DOI:** 10.1002/ehf2.12744

**Published:** 2020-06-13

**Authors:** Gerardo García‐Rivas, Elena Cristina Castillo, Adrian M. Gonzalez‐Gil, José Luis Maravillas‐Montero, Marion Brunck, Alejandro Torres‐Quintanilla, Leticia Elizondo‐Montemayor, Guillermo Torre‐Amione

**Affiliations:** ^1^ Tecnologico de Monterrey, Escuela de Medicina y Ciencias de la Salud, Cátedra de Cardiología y Medicina Vascular Monterrey Nuevo León Mexico; ^2^ Tecnologico de Monterrey, Hospital Zambrano Hellion, TecSalud, Centro de Investigación Biomédica San Pedro Garza García Nuevo León Mexico; ^3^ Red de Apoyo a la Investigación Universidad Nacional Autónoma de México and Instituto Nacional de Ciencias Médicas y Nutrición Salvador Zubirán Mexico City Mexico; ^4^ Tecnologico de Monterrey, School of Engineering and Science, FEMSA Biotechnology Center Monterrey Nuevo León Mexico; ^5^ Weill Cornell Medical College, Methodist DeBakey Heart & Vascular Center The Methodist Hospital Houston TX USA

**Keywords:** Heart failure, B cells, Inflammation, Therapeutics

## Abstract

Despite numerous demonstrations that the immune system is activated in heart failure, negatively affecting patients' outcomes, no definitive treatment strategy exists directed to modulate the immune system. In this review, we present the evidence that B cells contribute to the development of hypertrophy, inflammation, and maladaptive tissue remodelling. B cells produce antibodies that interfere with cardiomyocyte function, which culminates as the result of recruitment and activation of a variety of innate and structural cell populations, including neutrophils, macrophages, fibroblasts, and T cells. As B cells appear as active players in heart failure, we propose here novel immunomodulatory therapeutic strategies that target B cells and their products.

## Introduction

Heart failure (HF) is accompanied by a systemic pro‐inflammatory state,^1^ in which both the innate and adaptive immune system participate.^2^, ^3^ Despite mounting evidence linking inflammation and HF, specific immunomodulatory therapies for HF have not been successfully developed. This is explained in part because not all the components of the immune system have been thoroughly investigated in the context of HF, as is the specific role that each immune cell plays in the heart. Germane to this discussion is the fact that the accumulated evidence shows that B cells, both directly (by secreting antibodies) and indirectly (by antigen presentation and cytokines/chemokines secretion), play an essential role in the progression of HF. Therefore, we suggest that addressing B cells more than B‐cell products in HF patients may serve as therapeutic alternatives for patients with treatment‐refractory HF. In this review, we present current evidence of the role of B cells in adverse cardiac remodelling, highlighting that this role is independent of aetiology, and introduce our ongoing investigations into novel immunomodulatory therapeutic strategies that target B cells and their products.

## Antibody‐mediated mechanisms that contribute to cardiac dysfunction

Antibody‐mediated contribution to cardiac injury includes, on the one hand, direct consequences of anti‐cardiac antibodies binding to target cells and, on the other hand, the activation of the complement system following the formation of antigen–antibody complexes.

## Direct effects of anti‐cardiac antibodies

In a study of end‐stage failing human myocardium tissue, we reported the presence of immunoglobulin G (IgG) deposits in up to 70% of heart tissue evaluated. Approximately 50% of biopsies stained positive for the IgG of the type 3 subclass, and a smaller proportion was also positive for C3c deposition.^4^ As the IgG3 subclass exhibit the most effective complement‐fixating activity,^5^ its presence in heart tissue can provide powerful signals of cell injury and to recruit inflammatory cells. Remarkably, the presence of IgG3 and C3c in the myocardium correlated significantly with the length and severity of illness.^4^, ^6^ The evidence provided by this large cohort and others demonstrates a strong association of failing myocardium with B‐cell activation and potentially B cell‐mediated injury in HF in humans.^4^, ^6^, ^7^ An experimental model of ischaemic cardiac injury further supports the role of cardiac autoantibodies.^8^ In this model, ischaemic injury in control mice produced myocardial infarction (MI) and depressed ejection fraction, while infarct size was reduced, with cardiac function improved in Ig‐deficient mice.^8^

**Figure 1:**
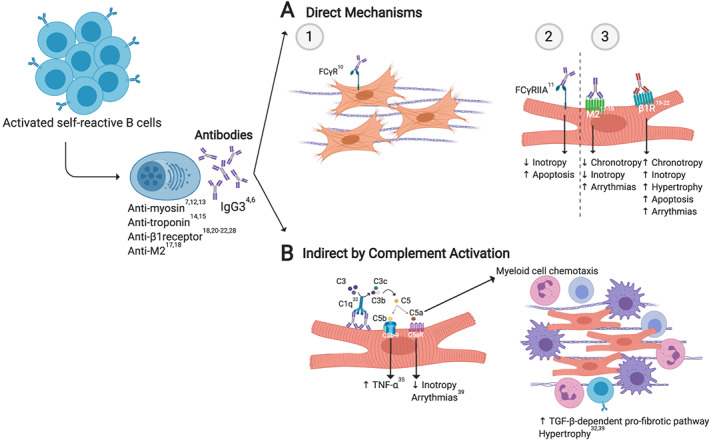
Antibody‐dependent mechanisms. (A) IgG3 antibodies can exert their function by being recognized through their Fc by Fc (fragment crystallizable region) γ‐receptor (FCγR) (1, 2) or by binding to specific surface receptors influencing their activity, as occurs with M_2_‐adrenergic and β_1_‐adrenergic receptors (3). (B) Antibody‐mediated disease may also induce the activation of the complement system via the classical pathway ending in membrane attack complex (C5b‐9) formation and chemotaxis of myeloid cells, allowing inflammation, fibrosis, and tissue dysfunction promoting hypertrophy and arrhythmias. C5aR, C5a receptor; TNF‐α, tumour necrosis factor‐α; TGF‐β, transforming growth factor‐β. Original image created with BioRender®.

Autoantibodies might cause injury either by directly interacting with target receptors on heart cells or by triggering signals via their Fc‐binding domain when it is interacting with Fc γ‐receptors (FcγR) present on the surface of a variety of cells.^9^ Activation of FcγR via the antibody Fc‐binding domain implies that antibody specificity is irrelevant for this pathway. Therefore, a large proportion of, or perhaps all, anti‐cardiac antibodies have these potential effects. FcγRs are present on cardiac fibroblasts^10^ and cardiomyocytes.^11^ FcγR signal transduction promotes fibrosis in cardiomyocytes and reduced calcium transients, cell shortening, and induction of cardiac cell death through activation of apoptotic pathways in myocytes^8^, ^10^, ^11^ (*Figure* *1* *A*).

Autoantibodies target a variety of proteins. In the context of HF, most identified antigens are present on the cell surface. However, a smaller number of intracellular proteins are also known as anti‐cardiac antibodies targets, such as sarcomere proteins (e.g. actin, myosin, and troponin).^7^, ^12^, ^13^, ^14^, ^15^ The consequences of anti‐cardiac antibodies presence on heart physiology depend on their target and also on how they modulate activity (*Table* *1*). For instance, the most extensively studied antibodies are those that target G protein‐coupled membrane receptors.^28^ Anti‐M_2_ receptor antibodies have been associated with negative chronotropism at the sinoatrial level,^23^, ^24^ negative inotropism,^29^ and supraventricular arrythmias.^24^ In contrast, antibodies specific to the β_1_‐adrenergic receptor induce positive chronotropism and inotropism,^20^, ^29^ cardiac hypertrophy, desensitization to catecholamines,^21^ and cardiomyocyte apoptosis.^22^ Clinically, anti‐β‐receptor antibodies are the best characterized and are associated with compromised left ventricular function,^30^ increased incidence of ventricular arrythmias,^21^, ^23^ and mortality in patients with HF.^21^, ^31^ (*Figure* *1* *A*).

**Table 1:** The specificity of cardiac autoantibodies identified in patients with HF

Antibody specificity	Aetiology	Findings	Reference
Heart mitochondria: M7 (sarcosine dehydrogenase)	DCM, HCM, and acute myocarditis	React with heart mitochondria	Klein *et al*.^16^
Laminin	DCM and myocarditis	Unknown	Wolff *et al*.^17^
Hsp60	DCM and AMI	Unknown	Latif *et al*.^7^
Actin, tropomyosin, and myosin light chain	DCM	Unknown	Latif *et al*.^7^
Adenine nucleotide translocator	DCM	Cytotoxic damage and enhanced calcium current in cardiac myocytes	Liao *et al*.^18^
β_1_‐ARs	DCM	Related to ventricular arrhythmias and sudden death, altering calcium management, modifying action potential, and apoptotic cell death	Liao *et al*.^19^; Christ *et al*.^20^; Iwata *et al*.^21^; Jane‐wit *et al*.^22^; and Chiale *et al*.^23^
M_2_‐muscarinic acetylcholine receptors	DCM	Play a role in mediating the development of atrial fibrillation probably by a sinus node dysfunction	Baba *et al*.^24^; Chiale *et al*.^23^
Sarcolemmal Na‐K‐ATPase	DCM	Associated to ventricular tachycardia	Baba *et al*.^25^
Cardiac myosin	DCM and children myocarditis	Impair myocyte contractility and suggest being associated with protein kinase A activation and non‐recovery	Warraich *et al*.^12^; Simpson *et al*.^13^
cTnI	DCM and AMI	Less ventricular function after acute myocardial infarction than patients with negative titres and associated with improved survival in patients with chronic DCM, but not ICM	Leuschner *et al*.^14^; Doesch *et al*.^15^
KChIP2	DCM and AMI	Associates with cell death on *in vitro* assays	Landsberger *et al*.^26^
ATP synthase β‐subunit	End‐stage HF	Unknown	Youker *et al*.^4^
CS	End‐stage HF	IgG CS autoantibodies in transplanted hearts of patients vs. natural IgM autoantibodies in healthy controls	Petrohai *et al*.^27^

DCM, dilated cardiomyopathy; HCM, hypertrophic cardiomyopathy; AMI, acute myocardial infarction; β_1_‐ARs, β_1_‐adrenergic receptors; HF, heart failure; cTnI, cardiac troponin I; KChIP2, Kv channel‐interacting protein 2; CS, citrate synthase.

## Anti‐cardiac tissue antibodies as activators of complement

Activation of the complement system is a well described outcome of the presence of antibodies. The complement system is an integral part of the innate immune response activated in HF through three pathways. The classical pathway is mediated by IgG and IgM antibodies, while the mannose‐binding lectin pathway and the alternative pathway depend on properdin (positive activator of complement activation) and plasma factor D.^4^, ^32^, ^33^, ^34^ All three pathways lead to the activation of plasma proteins in a coordinated manner by forming an enzymatic complex requiring the sequential formation of protein fragments. Activated complement may exhibit three downstream consequences: (i) induction of leukocyte chemotaxis by anaphylatoxins (C3a and C5a) through their respective receptors (C3aR and C5aR); (ii) opsonization (C3b, iC3b, and C3d) to facilitate transport and removal of immune complexes; and (iii) formation of the terminal membrane attack complex (C5b‐9) to directly lyse targeted cells^32^ (*Figure* *1* *B*).

As mentioned earlier, we have evidenced the presence of C3c in the myocardium, which correlated with the duration and severity of illness.^4^ In a different study, HF patients exhibited increased circulating levels of the cleavage end product of complement activation C5b‐9 compared with healthy controls, and this similarly associated with severity. C5b‐9 induced tumour necrosis factor‐α (TNF‐α) expression in cardiomyocytes,^35^ a cytokine known to contribute to cardiomyocyte hypertrophy, cardiac fibrosis, and apoptosis, all of which are critical components of injury in HF.^36^, ^37^ Interestingly, C5b‐9 deposits were associated with IgG deposition and TNF‐α expression in failing myocardium of patients with dilated cardiomyopathy (DCM).^35^

The anaphylatoxin C5a also plays a direct role in inotropic dysfunction via C5aR‐mediated signalling in cardiomyocytes, as evidenced in murine sepsis models.^38^ C5a appears to have an essential role in adverse cardiac remodelling, as C5aR antagonism decreased cardiac hypertrophy and perivascular fibrosis in a murine model of hypertension.^39^ Additionally, C5a is a potent chemokine that attracts myeloid cells to sites of damage^32^ and is capable of activating TGF‐β‐dependent pro‐fibrotic pathways in the heart^39^ (*Figure* *1* *B*).

## Triggers of B‐cell activation and anti‐cardiac antibodies in heart failure

About 10% of B cells are present in healthy hearts, as demonstrated in various mouse models.^40^, ^41^, ^42^ There, B cells are involved in modulating the myocardial immune cell traffic as well as left ventricular structure and function.^42^ Similarly, in patients with failing heart tissue, B cells are present in the intravasculature and in close contact with the endothelium.^42^ Following cardiac damage, damage‐associated molecular patterns (DAMPs) are released from damaged cardiac cells, interacting with antigen‐presenting cells such as B cells.^2^, ^43^ Therefore, B cells have an important role in cardiac tissue and can undergo DAMP‐mediated activation, which in turn activates T cells, overall contributing to the pro‐inflammatory milieu. In mouse cardiac tissue, B cells are present in the same proportion as neutrophils.^41^ Neutrophils are the leading infiltrating cells during MI^2^, ^44^ and are the most abundant cells in peripheral blood counts of patients along with the progression of ischaemic HF.^45^ It has been reported that B cells and neutrophils act cooperatively,^46^, ^47^ allowing an antibody response,^46^ but B cell‐helper neutrophil interactions in the heart remain to be studied. However, there are at least three mechanisms for the formation of anti‐cardiac cell autoantibodies. First, autoreactive naïve B cells evade negative selection mechanisms in the bone marrow, which then capture, process, and present cardiac antigens (cAgs) through major histocompatibility complex‐II molecules to activate autoreactive T helper cells.^48^, ^49^ Second, large antigens with repetitive sequences can generate a T cell‐independent humoral response, a mechanism previously proposed for the formation of myosin autoantibodies.^50^ Third, memory B cells could be activated by contact with low doses of cAgs re‐encounters, causing T‐cell activation and their differentiation to long‐lived antibody‐producing plasma cells.^51^ This final mechanism is particularly relevant because it would allow maintaining a constant autoantibodies production. All of these findings are consistent with the clinical observation that a higher number of B cells, naïve and memory, during an ST‐segment elevation MI (STEMI), are associated with increased mortality.^52^

Although the presence of self‐reactive lymphocytes is a prerequisite for the development of autoimmunity, the presence of inflammatory milieu is necessary to avoid peripheral tolerance while promoting autoreactive cell full activation.^53^ HF can be thought as chronic inflammatory state,^1^, ^54^ in which increased levels of pro‐inflammatory cytokines trigger immune activation upon cAgs encounter. This inflammatory state becomes more severe in terminal stages of the disease, as TNF‐α levels increase substantially with advancing New York Heart Association (NYHA) stages. The rise in TNF‐α levels is explained as TNF‐α is released from the failing myocardium into circulation.^37^, ^55^ Chronic inflammation is further promoted by peripheral nuclear factor‐κB activation secondary to widespread tissue hypoxia and free radical generation in advanced HF.^54^ This concept has been documented previously, in the context of diabetes mellitus type 1, where increased local concentrations of TNF‐α in pancreatic islets resulted in enhanced T‐cell autoreactivity to β cells.^56^ Furthermore, considering that TNF‐α is produced in Th1 inflammatory reactions, the increase in Th1:Th2 ratio is associated with adverse cardiac remodelling and impaired function following MI^57^ and in decompensated HF.^58^ This is consistent with results showing that peripheral Th cells are associated with left ventricular dysfunction.^59^ Interestingly, interferon‐γ (IFN‐γ) was significantly elevated in patients with NYHA III–IV compared with NYHA I–II patients 30 days after MI.^57^ Th1 response facilitates the activation of B cells.^60^, ^61^ IFN‐γ induces IgG3 expression,^62^ which is consistent with IgG3 deposits in failing myocardium.^4^, ^6^ Finally, IgG3 deposits in patients failing heart tissue are accompanied by mixed inflammatory infiltrates of B cells, T cells, and macrophages.^6^

Cardiac antigens may be a consequence of the expression of neo‐antigens and/or the failure of the mechanisms of self‐tolerance post‐cardiac injury. However, there is evidence of cross‐reactivity from antibodies towards non‐cardiac proteins, which also interact with the myocardium and alter its function. A well‐documented example is the case of anti‐Sjögren's syndrome, where related antigen A (anti‐SSA or Ro) antibodies are typically present in autoimmune disorders like Sjögren's syndrome and systemic lupus erythematosus (SLE). These antibodies can cross‐react with both T‐type Ca^2+^ channels (CaV3.1 and CaV3.2) and L‐type Ca^2+^ channels (CaV1.2 and CaV1.3), causing conduction disorders such as sinus bradycardia and atrioventricular block.^63^ Similar observations have been documented for autoantibodies recognizing the NaV1.5 sodium channel and those targeting the KV11.1, KV1.4, or KV7.1 potassium channels.^63^ This alternative mechanism is not necessarily caused by autoreactivity, as it can occur in response to infection, which generates antibody‐mediated cardiac injury by molecular mimicry. For instance, in patients with rheumatic heart disease, antibodies against streptococcal *N*‐acetylglucosamine and α‐helical coiled‐coil M proteins cross‐react with cardiac myosin and can produce myocardial damage.^64^

In patients with HF, mechanisms involving both autoreactivity and molecular mimicry may be taking place following exposure to antigens that are normally ‘hidden' from the immune system (as occurs in myocardial injury from any cause), a context in which autoantibodies, as well as other cellular and soluble inflammatory mediators, may arise and cause damage.^28^

## Antibody‐independent mechanisms

B cells can alter the cardiac function and induce remodelling by secreting factors that influence cardiomyocytes, cardiac fibroblasts, and leukocytes. B‐cell depletion in mouse models of HF resulted in a decrease in infarct size, adverse ventricular remodelling, and protected ventricular function,^65^, ^66^ as well as an attenuated hypertensive response.^67^ The protective consequences on B‐cell depletion were not recapitulated when T cells were eliminated in this model.^66^ B‐cell depletion was accompanied by a significantly decrease in TNF‐α, interleukin (IL)‐1β, IL‐18, and BNP serum levels, myocardium apoptosis, and IgG depositions^65^, ^66^, ^67^ in which the pro‐inflammatory, pathological phenotype was restored when B cells were reintroduced.^66^, ^67^

A recent report demonstrated that cardiac fibrosis was dependent on the direct modulation of a specific cardiac B‐cell subset (CD19^+^CD11b^−^).^40^ In this mouse model of HF, it was previously demonstrated that the splenic plasma cells (CD19^+^CD138^+^) and activated B cells (CD19^+^CD86^+^) are increased,^67^ which could migrate to the heart.^42^ Activated B cell secretes cytokines and chemokines that may be directly involved in pathways that lead to adverse cardiac remodelling such as increased recruitment of monocytes, higher differentiation of pro‐fibrotic macrophages, and increased expression of TGF‐β, collagen‐I, and IL‐1β by fibroblast and macrophages.^65^, ^67^ Activated B cells recruited inflammatory monocytes (Ly6C^+^) to the myocardium in a CCL7‐dependent fashion contributing to adverse ventricular remodelling^65^ that is impaired by B cell‐activating factor (BAFF) neutralization, which promotes B‐cell depletion. Patients' CCL‐7 serum levels positively correlated with increased risk of death and recurrent infarctions after acute MI.^65^ Therefore, systemic depletion of B cells is likely to reduce macrophage‐induced myocardial damage. As antigen‐presenting cells, activated B cells can activate CD4^+^ T cells and promote their differentiation into the Th1 phenotype. In turn, Th1 cells may stimulate cardiac fibrosis through direct cell‐to‐cell interaction with cardiac fibroblasts, favouring their transition to TGF‐β and collagen‐producing myofibroblasts.^68^ Consequently, B cells promote a full expression of HF and downstream cardiac injury by mediating immune cells chemotaxis and activation, and these are impaired in the absence of B cells.

**Figure 2:**
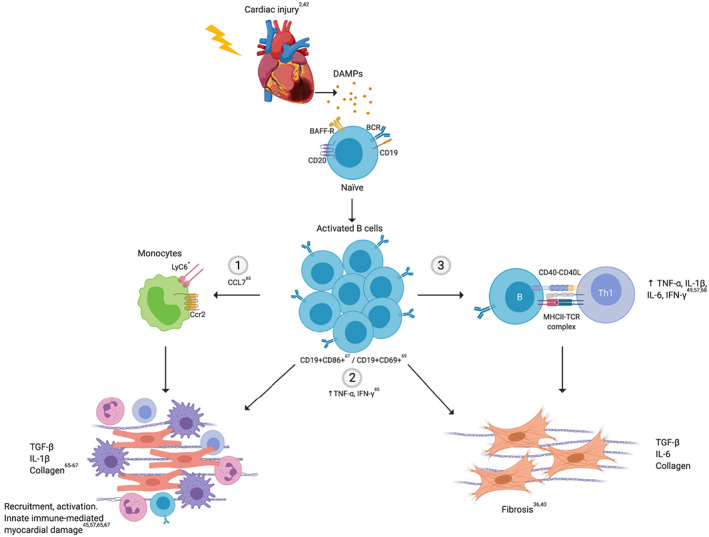
Antibody‐independent mechanisms. After cardiac ischaemic and non‐ischaemic injury, B cells become activated and proliferate in response to damage‐associated molecular patterns (DAMPs) that are released (from damaged cells and tissues) in response to cardiac injury. Their activation has been associated with chemotaxis of LyC6 + CCR2^+^ monocytes, which are involved in pathogenic remodelling and inflammation (1), though CCL7. The secretion of pro‐inflammatory cytokines is associated with fibrosis and detrimental function (2) as well as with proper cell activation and cell differentiation. The promotion of T‐cell activation and differentiation to the Th1 phenotype might be mediated by antigen cell presentation by B cells. This response contributes to the inflammatory milieu and may subsequently stimulate cardiac fibrosis through cardiac fibroblasts (3). BAFF‐R, B cell‐activating factor receptor; BCR, B cell receptor; CCL7, C–C motif chemokine ligand 7; CCR2, C–C chemokine receptor type 2; CD, cluster of differentiation (CD19 and CD20); IFN‐γ, interferon‐γ; IL‐1β, interleukin‐1β; Th1, type 1 helper T cell; TGF‐β, transforming growth factor‐β; TNF‐α, tumour necrosis factor‐α. Original image created with BioRender®.

B cells undergo activation following acute decompensation of HF, as indicated by the increased expression of CD69 in patients.^69^ Higher concentrations of BAFF correlated with increased risk of death or reinfarction.^65^ Furthermore, TNF‐α‐secreting B cells in patients with DCM are associated with enhanced cardiac fibrosis, as demonstrated by late enhancement on cardiac magnetic resonance imaging and higher levels of serum pro‐collagen type III.^36^ Thus, B cells may also directly participate in cardiac remodelling through up‐regulation of TGF‐β and IL‐6 and further maintenance of a detrimental inflammatory environment via TNF‐α, IL‐1β, and IL‐6 production (*Figure* *2*).

**Figure 3:**
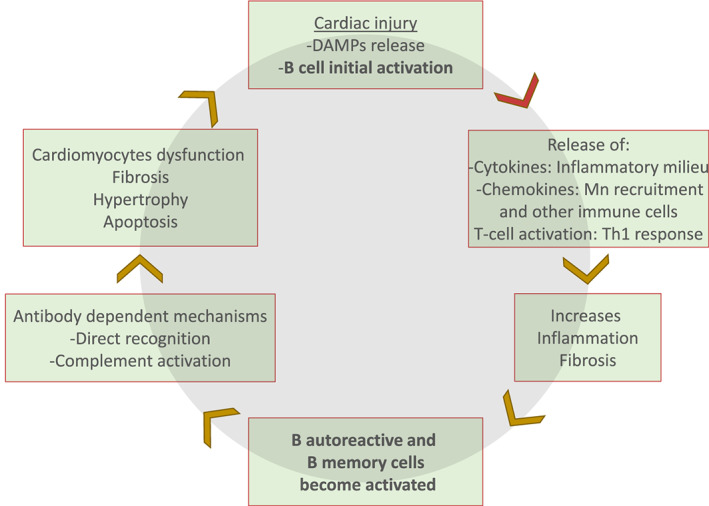
B cells play a central role in heart failure. After the cardiac injury, damage‐associated molecular patterns (DAMPs) are recognized, processed, and presented by resident B cells that become activated in a T‐dependent mechanism. In response, B cells secrete cytokines and chemokines that contribute with the inflammatory milieu along with the activated T cells mainly by a Th1 response (tumour necrosis factor‐α and interferon‐γ). Cell recruitment as monocytes (Mn) become activated, promoting fibrosis, hypertrophy, and tissue remodelling. The inflammatory milieu allows autoreactive B cells to become fully activated and differentiated into a memory B cell or plasma cell that produces mainly IgG3 antibodies against cardiac proteins. This induces further myocardial damage by antibody‐dependent mechanisms.

Altogether, B cells initiate a self‐perpetuating cycle of cardiac injury through antibody‐independent and antibody‐dependent mechanisms (*Figure* *3*), in which damage causes leakage of intracellular proteins and activation of self‐reactive B cells against other myocardial components. This damage is further exacerbated by the phenomenon of epitope spreading.^70^ Cardiac autoantigens are ubiquitously present in the myocardium and, as such, cannot be fully cleared by the ensuing inflammatory reaction. Therefore, it seems logical that the inflammatory response continues indefinitely and is amplified with every new insult to the myocardium (e.g. a new infarct or acute decompensation of HF). Accordingly, antibody and complement deposits tend to be more frequent in the late stages of HF.^4^ Importantly, this may partially explain why HF tends to follow an adverse natural history independently of current treatment strategies, which only target neuroendocrine components of the disease.

## Potential endogenous modulators of B‐cell response in heart failure

There are at least two possible mechanisms that regulate B‐cell function in the HF setting. The interaction of the neuroendocrine system and immune cells and the role of regulatory B and T cells (Bregs and Tregs, respectively).

First, both T and B cells express β_2_‐adrenergic receptors,^71^ and activation of T and B cells in the presence of norepinephrine enhances cytokine and IgG secretion, respectively.^72^ This implies that high levels of catecholamines may exacerbate immune activation in HF. In accordance, B cells from patients with congestive HF display intracellular Ca^2+^ leak due to ryanodine receptor (RyR1) phosphorylation, possibly as consequence of catecholamine overproduction, although their functional role in this context has not been evaluated, intracellular Ca^2+^ is fundamental to B‐cell activation.^73^ Interestingly, it has also been shown that catecholamines enhance monocyte mobilization from the bone marrow following MI,^74^ which may be synergistic with B cell‐derived CCL7 in generating myocardial damage^65^ and promoting vascular disease.

Second, Bregs have a role in suppressing self‐reactive B cells in various autoimmune diseases, and dysregulation of Bregs has been proposed as a mechanism of autoimmunity. Although specific surface marker or transcription factor profiles for Bregs subsets have not been clearly defined, these cells are primarily characterized by the expression of the anti‐inflammatory cytokine IL‐10 and the induced suppression of T‐cell responses, including the impairment of cytokine secretion by Th1 and Th17 lymphocytes.^75^, ^76^ This implies that Bregs may protect against contractile dysfunction by interfering with the pro‐inflammatory environment. Consistently, decreased levels of IL‐10 have worsened cardiac remodelling in murine models of angiotensin II‐induced cardiac injury.^77^ However, evidence of the role of Bregs in human HF has only been addressed by two contradictory reports. The first study showed increased numbers of circulating Bregs (CD19^+^CD5^+^CD1d^+^IL10^+^) in patients with DCM.^78^ The second study reported opposite results, albeit in a different Breg cell subpopulation (CD19^+^CD24^hi^CD27^+^IL10^+^), which also showed a decreased potential to suppress the TNF‐α production by T cells.^79^ Therefore, further studies to evaluate the role of Bregs in HF are warranted.

Regulatory T cells can regulate the activity of autoreactive B cells by two mechanisms: first, avoiding autoreactive B cells to become activated. Tregs have direct actions on B cells by inhibiting immunoglobulin class switching, inducing apoptosis,^80^ and inhibiting their activation and proliferation via interaction between programmed death‐1 and programmed death‐1 ligand present on the surface of Tregs.^81^ This could partially explain why lower circulating Tregs are associated with left ventricular dysfunction and poor prognosis in patients with HF,^82^, ^83^ as supported by the evidence in murine models of lupus, in which depletion of Tregs increases the production of autoantibodies while their administration has the opposite effect.^84^ Second, Tregs can also acquire an inflammatory phenotype and contribute to adverse myocardial remodelling, as was shown in a murine model of ischaemic cardiomyopathy, by increasing the expression of IFN‐γ and TNF‐α.^85^ This could create a permissive environment for autoreactive B cells to become activated to produce pro‐inflammatory cytokines and autoantibodies that exacerbate the initial insult. This evidence indicates that Tregs' interaction with B cells needs to be evaluated as Tregs could also represent a potential target for immunomodulatory therapies in HF.

## B cells as a therapeutic target in heart failure

Immunomodulatory strategies for HF to date have mostly targeted autoantibodies and pro‐inflammatory cytokines and have produced inconclusive results with subtle condition improvements only. These strategies include selective inhibition of TNF‐α, immunoadsorption, intravenous immunoglobulin, therapeutic plasma exchange, and non‐specific immunomodulation with autologous apoptotic cells, which are all reviewed in detail elsewhere.^43^, ^55^, ^86^

The reasons why these therapeutic approaches have not resulted in definite clinical improvement remain elusive, but some hypotheses in which B cells are involved can be drawn. TNF‐α blockers have been associated with worsening HF and mortality. Although interventions decreased levels of TNF‐α, myocardial function did not improve.^87^, ^88^ It was previously observed that treatment with anti‐TNF‐α blockers alters the distribution of peripheral blood B cells by increasing the frequency of pre‐switch IgD^+^CD27^+^ memory B cells in non‐responder patients with rheumatoid arthritis (RA).^89^ This could favour cAgs recognition by these cells and results in their differentiation to antibody‐producing plasma cells. In addition, it is worth noting the signalling amplification of the immune activation that occurs in HF, in which many other pro‐inflammatory cytokines and mediators, not just TNF‐α (such as IL‐1β and IL‐6), are responsible for continued myocardial damage.^1^ These other mediators, either directly, by inducing cardiac dysfunction, or indirectly, by activating effector functions of various classes of immune cells, including B cells, can also promote the progression of HF.

On the other hand, immunoadsorption, plasma exchange, and intravenous immunoglobulin, which non‐specifically deplete antibodies from the patient, have been associated mostly with significant improvements in cardiac function, in the short term.^69^, ^90^, ^91^ However, the long‐term benefit is unclear, as cardiac autoantibodies reappear in a proportion of treated patients who eventually may require a heart transplant or a ventricular assist device.^91^ This is consistent with the fact that these therapies target the damage‐mediating product but not its source. Indeed, eventual disease relapse may be related to the reappearance of anti‐cardiac antibodies produced by terminally differentiated B cells. This prompted the non‐specific immunomodulation strategy employed in the ACCLAIM trial, in which apoptotic autologous cells were injected to patients with the rationale that apoptotic cells would induce a systemic anti‐inflammatory response by polarizing phagocytic cells to an alternatively activated phenotype, promoting decreased inflammatory myocardial damage.^92^ However, only a small subset of patients with non‐ischaemic HF demonstrated significant benefit.^92^ It has been observed that some patients with chronic inflammation had impaired clearance of apoptotic cells that eventually leads to secondary necrosis and damage.^93^

Novel strategies targeting other immune components are currently undergoing evaluation in clinical trials.^94^ Here, considering the evidence described earlier, we discuss and evaluate the possibility of new treatment strategies that directly target B cells in HF.

Rituximab (RTX) is a chimeric monoclonal antibody that targets CD20, a membrane‐spanning protein present exclusively in the majority of B lineage cells (except pro‐B cells and plasma cells).^95^ RTX exerts its B cell‐depleting action by binding to CD20 molecules on the B cell's membrane, activating the classical pathway of the complement system, facilitating cell‐mediated cytotoxicity and apoptosis.^96^ Clinical experience with RTX is extensive, and it is currently approved for the treatment of haematological malignancies and prototypical autoimmune diseases, such as RA.^51^ Although RTX clears virtually all CD20^+^ B cells from peripheral blood, B cells residing in secondary and tertiary lymphoid organs may survive depletion.^95^, ^97^ Notwithstanding, repopulation with beneficial B‐cell subsets may follow treatment with RTX. For instance, patients with thrombotic thrombocytopenic purpura treated with RTX demonstrated slow regeneration of memory B‐cell subsets, and BAFF‐R expression was reduced in all B‐cell subsets after RTX.^98^ As BAFF is a crucial survival signal for B cells, the decreased in BAFF‐R expression could potentially hinder autoreactive B‐cell maturation in the periphery and hence could be a long‐lasting indirect benefit of RTX in HF patients. After RTX, B‐cell repopulation may also be characterized by higher proportions of IL‐10‐producing Bregs,^51^ which may also benefit patients with HF. However, experience with the drug has revealed potential adverse effects and variable responses between patients, as well, differential susceptibility to RTX‐mediated depletion within B‐cell subpopulations.^95^

Thus, the use of RTX as a therapeutic agent in HF could lead to a decrease in circulating cardiac autoantibodies, B cell‐derived cytokines, and activation of self‐reactive T cells, which jointly could potentially prevent further progression of HF.

Tschöpe *et al*. ^99^ have recently published a case series of six patients with refractory inflammatory DCM treated with RTX. Five patients had a favourable response to RTX, as indicated by decreased NYHA functional class at 8 weeks of follow‐up, improved left ventricular ejection fraction, and decreased B‐cell infiltrates in endomyocardial biopsies. However, one patient did not improve: NYHA class did not change, left ventricular ejection fraction remained significantly compromised, and, paradoxically, infiltrated B cells in the myocardium nearly doubled. Although the authors did not propose an explanation to this non‐responder phenotype, we hypothesize that it may be related to an unfavourable B‐cell subset that is resistant to depletion by RTX, as was described in patients with RA.

Our group designed a phase II, single‐centred prospective clinical trial. We propose that RTX may be safely used as adjunctive therapy in the management of treatment‐refractory HF. Our ongoing study includes patients with ejection fraction ≤40%, NYHA functional class III/IV, who are unresponsive to standard HF treatment and have not previously been treated with an immunosuppressive drug. RTX dose will mimic that already applied to post‐transplant patients and patients with RA, based on prior evidence of safety.^100^

After RTX, B‐cell repopulation may also be characterized by higher proportions of IL‐10‐producing Bregs,^51^ which may also benefit patients with HF. However, experience with RTX has revealed potential adverse effects and variable responses between patients, as well as differential susceptibility to RTX‐mediated depletion within B‐cell subpopulations.^95^

The use of BAFF antagonists may be another therapeutic possibility in HF. The BAFF‐directed antibody belimumab is currently approved for treatment‐resistant SLE in adults, reducing flares and overall disease activity when used in combination with standard therapy.^101^ However, it is known that belimumab only partially inhibits the production of IgG autoantibodies, as it depletes both naïve and activated B cells, but not memory B cells.^51^ Considering that pathogenic autoantibodies in HF are mostly of the IgG3 class and that B‐cell subsets in this context are largely unknown, further research is necessary to justify its use in HF.

There are other B cell‐target therapies, but they are less extensively studied, only on patients with lymphoma or one autoimmune disease (SLE and RA), with recent available data (2018).^102^ A comparative study between RTX and abatacept, a B–T cell co‐stimulatory inhibitor, showed that RTX had better outcomes in terms of failure defined as all cause death.^103^

## Determination of B‐cell subsets in heart failure patients

The strategies mentioned earlier exhibit varied depletion efficacy depending on B‐cell subsets.^95^, ^101^ If B cell‐depleting strategies are to be used for the treatment of HF, then the characterization of B‐cell profiles, as has been determined in the setting of prototypical autoimmune diseases,^101^ could be useful from a clinical standpoint.

First, it would help predict which subsets of patients are more likely to respond to treatment or have a sustained clinical response, a concept supported by studies in RA in which patients who had higher proportions of double‐negative naïve B cells (CD19^+^IgD^−^CD27^−^) and lower percentages of plasmablasts (CD19^+^IgD^+^CD27^++^) and memory B cells (CD19^+^IgD^+^CD27^+^CD95^−^; CD19^+^CD27^+^) were more likely to have a favourable clinical response with RTX.^104^, ^105^ Furthermore, the clinical response in RA patients treated with RTX correlated positively with the depletion of the pre‐switch memory B cells (CD19^+^IgD^+^CD27^+^CD95^−^), which is the increased cell population in response to anti‐TNF‐α therapy.^89^ These findings suggest that patients with higher numbers of memory B cells or plasmablasts that might correspond to those with increased disease activity are more likely to be resistant to therapy. As discussed earlier, memory B cells are responsible for the swift humoral immune response that occurs upon re‐encounter with antigen.^51^ However, plasmablasts are short lived in peripheral blood before they home to bone marrow, mucosal tissues, or sites of ongoing inflammation.^50^ Therefore, their presence may imply current autoantigen presentation and B‐cell activation. In patients with HF, the relative abundance of these subsets could precipitate a detrimental feedback loop of myocardial injury, as described earlier. Supporting this concept, van den Hoogen *et al*. have recently reported that patients with HF had increased proportions of circulating plasmablasts and decreased transitional/regulatory B cells compared with healthy controls. Interestingly, stratified analysis revealed that these differences were more pronounced in patients with ischaemic HF, which likely reflects the nature of the insult to the myocardium.^6^

Ischaemic damage results in massive cardiomyocyte necrosis and leak of cAgs to the circulation, while non‐ischaemic insults result in increased wall stress with a less significant release of cAgs. Although the observation of increased plasmablasts did not reach statistical significance, this was likely due to small sample size (*n* = 10), as we have also identified increased circulating plasmablasts in 21 patients with HF (unpublished data). Furthermore, patients admitted for STEMI had significantly higher total B‐cell counts than no‐STEMI and control groups, in which naïve and memory B cells demonstrated a strong positive correlation with troponin I and creatine kinase levels (plasmablasts were not measured).^52^ Interestingly, the increased level of circulating memory B cells correlated with the 6 month probability of death from admission, as calculated with the GRACE score.^52^ The fact that STEMI, by definition, is indicative of cardiomyocyte death and the release of intracellular products and that increased B‐cell counts were observed in this group, together with the observation that patients with ischaemic HF tend to have higher levels of plasmablasts,^6^ further supports the involvement of B cells in myocardial damage, as previously reported in murine models.^65^

Furthermore, knowledge of the relative proportions of B‐cell subsets in HF may explain why most, but not all, end‐stage HF patients have antibody‐dependent‐mediated myocardial damage. Indeed, a considerable fraction of end‐stage HF patients lacks antibody or complement deposits in the myocardium.^4^, ^69^ These deposits being observed more often in more severe HF patients or longer time since diagnosis^4^ suggest that cumulative insults to the myocardium are bypassing peripheral tolerance mechanisms due to continued exposure of cardiac self‐antigens, resulting in higher proportions of pathogenic B cells. Hence, knowledge of which B‐cell subsets are altered throughout the spectrum of HF could be helpful to clarify this hypothesis.

Finally, evidence that specific B‐cell subsets cause disease progression would strongly support the claim for developing novel selective immunotherapies or treatments.

## Conclusions

Heart failure is characterized by a maladaptive process of cardiac interstitial fibrosis and contractile dysfunction, which may be initiated and maintained following insults to the myocardium. Among many processes that lead to HF, B cells are highlighted as playing a prominent role in its progression, regardless of aetiology, through mechanisms that are dependent and independent of antibody production. These mechanisms include secretion of pro‐inflammatory cytokines,^36^ monocyte recruitment following myocardial injury,^65^ and interaction with CD4^+^ T helper cells, with a subsequent amplification of the inflammatory cascade.^49^ On the other hand, the production of antibodies directed against cAgs,^4^ predominantly of the IgG3 subtype, may fix and activate complement to promote myocardial inflammation, injury, and remodelling.^4^, ^35^, ^38^, ^39^ Then, apoptosis^66^ and cardiac functional impairment alter cardiac contraction and heart rate^28^, ^29^ (*Figure* *3*). Additionally, regulatory T‐cell^82^ and B‐cell dysfunction^79^ may further enhance self‐reactive B‐cell responses in HF. Further research must confirm which B‐cell subpopulations may mediate continued myocardial damage in HF, as this would support trials of more selective therapeutics, possibly decreasing the scope of adverse events linked to the currently available therapies.

## Conflict of interest

None declared.

## Funding

This work was partially supported by the GIEE Medicina Cardiovascular y Metabolomica (Tecnologico de Monterrey, 0020209M01) as well as the Consejo Nacional de Ciencia y Tecnología (CONACYT) grants 151136, 133591, and 269399, and Fronteras de la Ciencia grant (0682). A.M.G.‐G. and A.T.‐Q. were supported by the Graduate Student Fellowship of CONACYT.
